# TREM2^+^ macrophages mediate anticancer therapy-induced immune evasion in lung adenocarcinoma via the CCL5/CCL22-Treg axis

**DOI:** 10.3389/fimmu.2026.1914462

**Published:** 2026-10-06

**Authors:** Chi Young Kim, Yoon Jin Cha, Min Kyung Park, Yoon Soo Chang

**Affiliations:** 1 Department of Internal Medicine, Yonsei University College of Medicine, Seoul, Republic of Korea; 2 Department of Pathology, Yonsei University College of Medicine, Seoul, Republic of Korea

**Keywords:** anticancer therapy, cisplatin, lung adenocarcinoma, osimertinib, regulatory T cells, single-cell RNA sequencing, TREM2, tumor-associated macrophages

## Abstract

**Introduction:**

Osimertinib and cisplatin are widely used as standard treatments for non-small cell lung cancer (NSCLC); however, their effects on tumor microenvironment remodeling remain poorly understood. This study investigated whether TREM2-positive tumor-associated macrophages (TAMs) promote immune evasion through the CCL5/CCL22–CCR5/CCR4–Treg axis under anticancer treatment.

**Methods:**

The spatial and quantitative correlations between TREM2^+^ and FOXP3^+^ cells were assessed using NSCLC patient-derived single-cell RNA sequencing (scRNA-seq) datasets, human lung adenocarcinoma tissue microarrays, and cisplatin-treated mouse lung cancer models. Mediators linking TREM2^+^ and FOXP3^+^ cells were identified by cell–cell communication analysis using CellChat and gene expression profiling of THP-1-derived macrophages treated with osimertinib or cisplatin, with validation in bone marrow–derived macrophages.

**Results:**

scRNA-seq dataset analysis showed significantly enriched TREM2^+^ and FOXP3^+^ cells in lung adenocarcinoma tissues compared with adjacent normal lung, with a significant positive correlation at the aggregated single-cell level (Spearman’s rho = 0.7323, P < 0.001). CCL5 and CCL22 were identified as key mediators linking TREM2^+^ TAMs and FOXP3^+^ Tregs and were markedly induced by treatment but suppressed by TREM2 knockdown or pharmacologic inhibition. TREM2 overexpression further enhanced their induction. Mechanistically, CCL22 induction was broadly dependent on SYK, Src, AKT, NF-κB, or STAT3 signaling pathways, whereas CCL5 upregulation depended on Src, NF-κB, and STAT3, with SYK involvement varying by cell type. In the orthotopic Lewis lung carcinoma (LLC) model, Trem2 inhibition reduced Treg infiltration and mitigated chemotherapy-associated immune evasion.

**Conclusion:**

These findings indicate that TREM2^+^ TAMs drive CCL5/CCL22-mediated Treg recruitment and support targeting TREM2 to enhance therapeutic efficacy of standard NSCLC treatments.

## 1 Introduction

Therapeutic options are limited for patients with advanced non-small cell lung cancer (NSCLC) who lack actionable mutations or have experienced failure of targeted therapies. For these patients, cisplatin-based doublet chemotherapy remains the most widely utilized regimen (1, 2). Osimertinib, a third-generation EGFR tyrosine kinase inhibitor (TKI), is the standard of care for patients with EGFR-mutated NSCLC (3). However, real-world clinical experience with osimertinib remains limited, particularly in combination therapy settings. The phase III FLAURA2 trial demonstrated improved outcomes with the combination of osimertinib and platinum-based chemotherapy compared with those of osimertinib monotherapy, supporting the emerging role of this regimen in clinical practice (4).

The tumor microenvironment (TME), consisting of tumor cells, immune cells, fibroblasts, blood vessels, and the extracellular matrix, plays a crucial role in tumor initiation, progression, and therapeutic response through dynamic interactions with cancer cells (5–8). Despite extensive research into the TME, therapy-induced alterations in the NSCLC TME are still largely unknown.

Within the TME of NSCLC, tumor-associated macrophages (TAMs) are heterogeneous and predominantly exhibit an M2-like immunosuppressive phenotype, contributing to tumor progression, metastasis, and therapy resistance (7, 9–12). Among various mechanisms, TAM-derived CCL22 promotes the recruitment of regulatory T cells (Tregs), thereby impairing anti-tumor immunity and correlating with poor clinical outcomes (13–15). Myeloid cells overexpressing TREM2 — hereafter referred to uniformly as TREM2^+^ TAMs — are CD68^+^ tumor-associated macrophages and a key component of the immunosuppressive TME (16). Additionally, TREM2^+^ TAMs suppress effector CD8^+^ T cell function by restricting proliferation and cytotoxic potential, while upregulating co-inhibitory receptors such as CTLA4 and TIM3 (16–18). TREM2 overexpression, typically associated with M2-type macrophages, is also implicated in resistance to immune checkpoint inhibitor therapy. Notably, combination therapy using TREM2-targeting antibodies alongside PD-1 inhibitors has been shown to enhance T cell infiltration and augment antitumor efficacy (19).

TREM2^+^ TAMs are more abundant in lung cancer tissues than in normal lung tissues. Cisplatin further increases the TREM2^+^ TAMs within tumor tissue. Furthermore, treating the THP-1 monocytic cells with anticancer agents (cisplatin and osimertinib) increases TREM2 expression, promotes M2 polarization, and alters phagocytic activity (20).

In this study, we aimed to investigate how TREM2^+^ TAMs shape the T cell population within the TME to identify the key mediators responsible for this interaction. To achieve this, we analyzed the spatial distribution and numerical correlations of TREM2^+^ TAMs and T/NK cell populations in both normal lung tissue and the NSCLC TME. Our analysis utilized a single-cell RNA sequencing (scRNA-seq) dataset, human lung adenocarcinoma tissue microarrays (TMAs), and a cisplatin-treated mouse lung cancer model. Through microarray analysis, we identified candidate mediators derived from TREM2^+^ TAMs that may influence T cell cluster dynamics. We further explored the regulatory mechanisms of these mediators using signaling pathway inhibitors. Finally, we validated the impact of Trem2 inhibition on the TME *in vivo* using mouse models undergoing chemotherapy. Collectively, this study proposed a targeted approach to modulate the immunosuppressive environment mediated by TREM2^+^ TAMs in the context of lung cancer treatment.

## 2 Materials and methods

### 2.1 Materials and study cases

Human THP-1 cells were purchased from the Korean Cell Line Bank (Seoul, Korea), and murine Lewis lung carcinoma (LLC) cells (Cat# CRL-1642; ATCC) were obtained from the American Type Culture Collection (Manassas, VA, USA). Other materials used in this study are listed in **Supplementary Table 1**.

### 2.2 Tissue microarray construction

In total, 116 cases of pathologically confirmed lung adenocarcinoma (2014–2018) were selected for constructing the TMA. The inclusion criteria were as follows: (1) curative lung resection performed, and (2) availability of representative FFPE tumor blocks. Patients who received neoadjuvant or adjuvant chemotherapy were excluded. Representative tumor areas were marked on H&E-stained slides, and 3 mm tissue cores were extracted and arranged into 6 × 5 recipient TMA blocks. Each separate tissue core was assigned a unique TMA location number that was linked to a database containing other clinicopathologic data. This study was approved by the Institutional Review Board (IRB # 4-2021-1206).

### 2.3 scRNA-seq dataset and analysis

The 10x Chromium-based scRNA-seq datasets for NSCLC were obtained from previously reported studies (21, 22). After quality control, dataset integration, and removal of malignant epithelial cells by inferCNV, cells were graph-clustered in Seurat and assigned to major lineages; the T/NK and myeloid compartments were then each re-clustered into subsets, following the annotation pipeline established for this cohort (20, 22). Within the myeloid compartment, monocyte, macrophage, and dendritic-cell states were resolved as separate subclusters, and TREM2-expressing cells were extracted (TREM2 > 2.5) and defined as an additional subcluster; on the annotated manifold these cells mapped predominantly to the CD163/LGMN macrophage subcluster. The complete workflow, marker sets, and clustering parameters are provided in the **Supplementary Methods** (“Identification and Annotation of Myeloid Subsets”), and all analysis code is openly available (see Data Availability). Intercellular interactions between TREM2^+^ TAMs and T/NK subclusters were estimated using the CellChat R package.

### 2.4 Macrophage culture and polarization

THP-1 cells were differentiated into M0 macrophages using 150 nM PMA (P1585, Sigma-Aldrich, St. Louis, MO, USA) for 24 h. Subsequent polarization into M1 (LPS/IFN-γ) or M2 (IL-4/IL-13) phenotypes was performed as detailed in the **Supplementary Methods**. Primary murine bone marrow–derived macrophages (BMDMs) were isolated from C57BL/6 mice and differentiated with 20 ng/mL M-CSF for 6 days.

### 2.5 Generation of *TREM2*-modified THP-1 cells

Stable *TREM2* knockdown was achieved using shRNA (HuSH-29™, Origene Technologies, Rockville, MD, USA) transfection with Lipofectamine 3000 (Invitrogen; Thermo Fisher Scientific, Inc., Carlsbad, CA, USA). For overexpression, THP-1 cells were transduced with *TREM2* lentiviral particles (Origene) at an MOI of 60 with 8 μg/mL Polybrene. For selection, cells were maintained with 0.5 μg/mL puromycin.

### 2.6 Microarray and RT-qPCR

Microarray profiling was performed using the GeneChip Human Gene 2.0 ST Array (Affymetrix; GEO accession **GSE309879**). For RT-qPCR, mRNA was quantified using TaqMan Fast Virus 1-Step Master Mix (Thermo Fisher Scientific). The target gene expression was normalized to that of *GAPDH* using the 2^-ΔΔCt^ method.

### 2.7 Immunoblotting

Cells were lysed in 2× LSL buffer; equal amounts of protein were separated using SDS-PAGE and transferred to PVDF membranes. Membranes were incubated with primary antibodies followed by HRP-conjugated secondary antibodies. Signals were detected using ECL substrate and quantified in ImageJ (v1.53g; NIH), with values normalized to those of β-actin.

### 2.8 Immunohistochemistry and image quantification

IHC and immunofluorescence (IF) staining were performed using the Dako REAL EnVision Detection System (Dako; Agilent Technologies, Inc., Santa Clara, CA, USA). High-resolution images were acquired using an LSM980 confocal microscope (ZEISS, Jena, Germany) and an AxioScan7 slide scanner (ZEISS), and subsequent image analysis was performed using QuPath (v0.5.1). A machine learning-based object classifier was used for automated cell classification (e.g., nuclear FOXP3^+^ or cytoplasmic TREM2^+^).

### 2.9 Mouse experiments

All procedures were approved by the IACUC (protocol 2025-0105) and followed the ARRIVE guidelines. Orthotopic tumors were established by injecting 1 × 10^5^ LLC cells (in 20 µl of PBS: Matrigel) into the left lung of 8-week-old C57BL/6 mice. After one week of stabilization, pharmacological treatments were initiated. Mice were euthanized for lung harvest at 8 days after the treatment initiation, or upon reaching humane endpoints (**Supplementary Figure 1**). All animal experiments were approved by the Institutional Animal Care and Use Committee of Yonsei University (protocol No. 2025-0105) and conducted in accordance with the ARRIVE guidelines and institutional animal care regulations.

### 2.10 Ethics approval statement

This study was approved by the Institutional Review Board of Yonsei University College of Medicine (IRB No. 4-2021-1206). Because this study used archived and anonymized human tissue samples, the requirement for informed consent was waived by the Institutional Review Board.

### 2.11 Statistical analysis

All tests were two-sided (α = 0.05). Baseline differences were assessed using Welch’s t-tests, and the combined effects of the pretreatment and drug were analyzed using two-way ANOVA (η^2p^ for effect size). Multiplicity was controlled using the Benjamini–Hochberg false discovery rate (FDR). scRNA-seq data were analyzed using the Wilcoxon rank-sum or signed-rank tests as appropriate.

## 3 Results

### 3.1 Annotation and distribution of T-cell populations

We performed unsupervised clustering using scRNA-seq datasets derived from paired normal-appearing lung and tumor tissues from two previous studies, and identified 10 T-cell–related subclusters (21, 22). Annotation proceeded stepwise by marker-gene analysis. First, we compared the expression of *CD3D, CD3E*, and *CD3G* between each candidate subcluster of the T/NK lineage and all other lineages in the lung TME, classifying clusters with a relatively low expression of these genes as NK cells. We then determined the differentially expressed genes (DEGs) across all 10 clusters, including the NK cluster, and annotated the remaining nine T-cell subclusters based on canonical markers. Expression of *TRAC, TRBC1, TRBC2, TRDC, TRGC1*, and *TRGC2* was used to distinguish αβ from γδ T cells: seven clusters with high *TRAC/TRBC1/TRBC2* were assigned to αβ T cells, and two clusters with high *TRDC/TRGC1/TRGC2* were assigned to γδ T cells. Next, *CD4, CD8A*, and *CD8B* expression separated the CD4^+^ and CD8^+^ subsets, with cells showing high *CCR7* and *SELL* defined as naïve T cells. Within CD4^+^ T cells, *FOXP3/ICOS*-high cells were annotated as Tregs, whereas clusters enriched for *CD40LG, IL7R*, integrins (*ITGAL, ITGAE, ITGA4*), and tissue-retention markers (*CD44, CD69*) were annotated as CD4_Trm. Within CD8^+^ T cells, clusters with high *GZMB, GZMH, GZMK*, and *PRF1* were annotated as CD8_effector, and those with characteristic *IL7R* and tissue-retention marker expression were annotated as CD8_Trm (**Figures 1A,B**). Comparing cluster distributions between normal and tumor tissue, five clusters—naïve T cells, Tregs, CD4_Trm, CD8_Teff_1, and CD8_Trm—were found to be significantly increased in tumors, whereas the CD8_Teff_3, γδT_1, and NK cells were significantly decreased (**Figures 1C, D**; **Supplementary Figure 2**).

**Figure 1:**
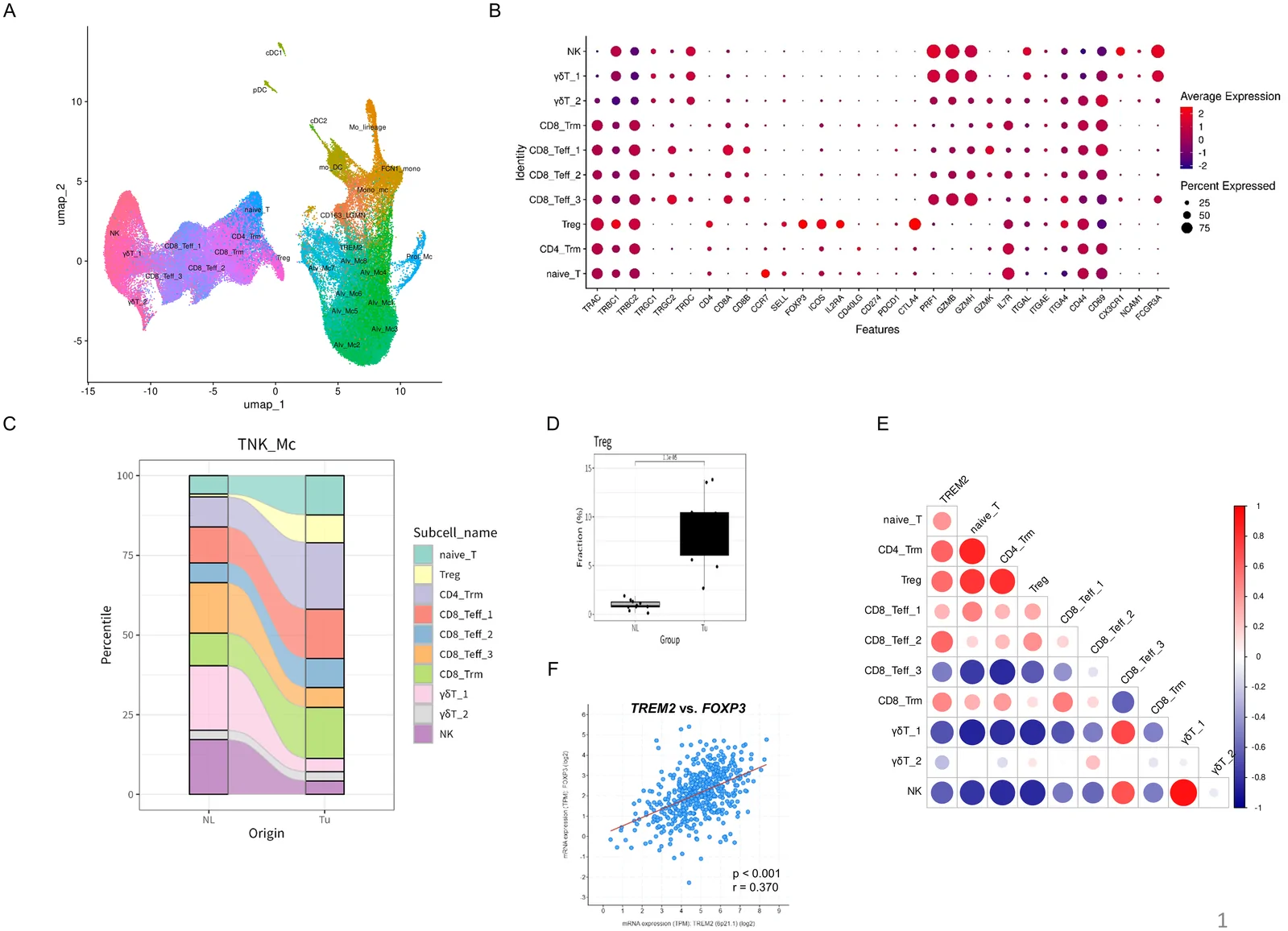
Distribution of TREM2^+^ TAMs and T/NK subclusters in scRNA-seq data from lung cancer (Tu) and adjacent normal-like lung (NL). **(A)** UMAP of T/NK and myeloid cell clusters derived from unsupervised clustering of the scRNA-seq dataset. **(B)** Dot plot showing the expression of representative marker genes used for cell-type annotation. **(C)** Alluvial plot showing the distribution of T/NK subclusters between Tu and NL. **(D)** Box plot comparing the proportion of the Treg subcluster between Tu and NL (P value calculated by Wilcoxon signed-rank test). **(E)** Dot correlation plot showing correlations in cell abundance between TREM2^+^ TAMs and the T/NK subclusters. **(F)** Scatter plot showing the association between *TREM2* and *FOXP3* expression in the LUAD cohort from cBioPortal (P value calculated by Spearman’s correlation test; r, Spearman’s rho).

In parallel, the myeloid compartment was re-clustered into 17 subclusters — four monocyte/differentiation states (Mo-lineage, FCN1-mono, Mono-Mc, CD163/LGMN), nine macrophage states (Alv-Mc1–8, Prol-Mc), and four dendritic-cell states (mo-DC, cDC1, cDC2, pDC) — each annotated by canonical markers (**Supplementary Methods**). Because the dendritic-cell (*CLEC9A, LAMP3, CD1C/FCER1A, CLEC4C*) and monocyte (*FCN1, S100A8, VCAN, CD300E*) lineages were resolved as their own clusters, TREM2 was confined to the differentiated macrophage compartment and was absent from dendritic-cell and pure-monocyte clusters. TREM2-high cells (TREM2 > 2.5) were therefore extracted and added as a distinct subcluster that mapped predominantly to the CD163/LGMN state, with a minor fraction on the lipid-associated Alv-Mc4/5 states — consistent with our prior characterization of this population in the same cohort (20, 22). This population co-expressed the macrophage/TAM markers *CD68, CD163, SPP1, LGMN, APOE*, and *GPNMB* while lacking alveolar-macrophage markers (*FABP4, PPARG, MARCO*), consistent with an M2-like, CD163^+^ tumor-associated macrophage state (20); its discriminating signature is given in **Supplementary Table 2**.

To assess whether the distribution of TREM2^+^ TAMs is linked to T-cell compartmentalization, we examined the numerical correlations between the TREM2^+^ TAMs and T/NK subclusters in the scRNA-seq dataset. The number of TREM2^+^ TAMs was positively correlated with Tregs and negatively correlated with NK cells (**Figure 1E**; **Table 1**). Finally, analysis of the TCGA-LUAD cohort in cBioPortal revealed a significant positive correlation between *TREM2* and *FOXP3* mRNA expression (r = 0.370, *P* < 0.001) (**Figure 1F**; **Supplementary Table 3**). Collectively, these findings indicate that enrichment of TREM2^+^ TAMs is associated with the accumulation of FOXP3^+^ cells in the TME, suggesting potential reciprocal interactions that support their co-enrichment.

**Table 1:** Correlation between TREM2^+^ TAMs and T/NK cell subclusters.

NK/T_subcluster	Spearman_rho	p_value	q_FDR(BH)
naive_T	0.6165	0.0038	0.0063
CD4_Trm	0.7398	0.0002	0.0006
Treg	0.7323	0.0002	0.0006
CD8_eff_1	0.2992	0.1999	0.2222
CD8_eff_2	0.4707	0.0362	0.0453
CD8_eff_3	-0.7759	0.0001	0.0005
CD8_Trm	0.5594	0.0103	0.0148
γδT_1	-0.7624	0.0001	0.0005
γδT_2	-0.2782	0.2350	0.2350
NK	-0.6707	0.0012	0.0024

### 3.2 Positive correlation between TREM2^+^ TAMs and FOXP3^+^ cells

We next evaluated whether the observed patterns could be applied to human tissues. We performed IHC for TREM2 and FOXP3 in TMAs comprising 116 treatment-naïve lung adenocarcinomas and quantified positive cells within the TME. TREM2^+^ TAM counts showed a modest but significant positive correlation with FOXP3^+^ cell counts (r = 0.199, P = 0.033), supporting a link between TREM2^+^ TAMs and putative Treg infiltration (**Figures 2A,C****;** **Table 2**; **Supplementary Figure 3**). In an LSL-Kras-G12D mouse lung cancer model, treatment with cisplatin increased both the Trem2^+^ and Foxp3^+^ cells relative to the vehicle, and these counts again showed a positive correlation (r = 0.249, *P* = 0.026**;** **Figures 2B,C**). These correlations were modest in magnitude, as expected in the heterogeneous TME. However, their consistent direction across human tissue, an independent mouse model, and the aggregated single-cell data (rho = 0.7323) — further supported by the categorical between-group differences reproduced in an independent cohort (20; **Figures 2D**, **3D, E** in that study) — indicates that TREM2^+^ TAM enrichment is consistently, though not tightly, associated with increased FOXP3^+^ cells in the TME.

**Figure 2:**
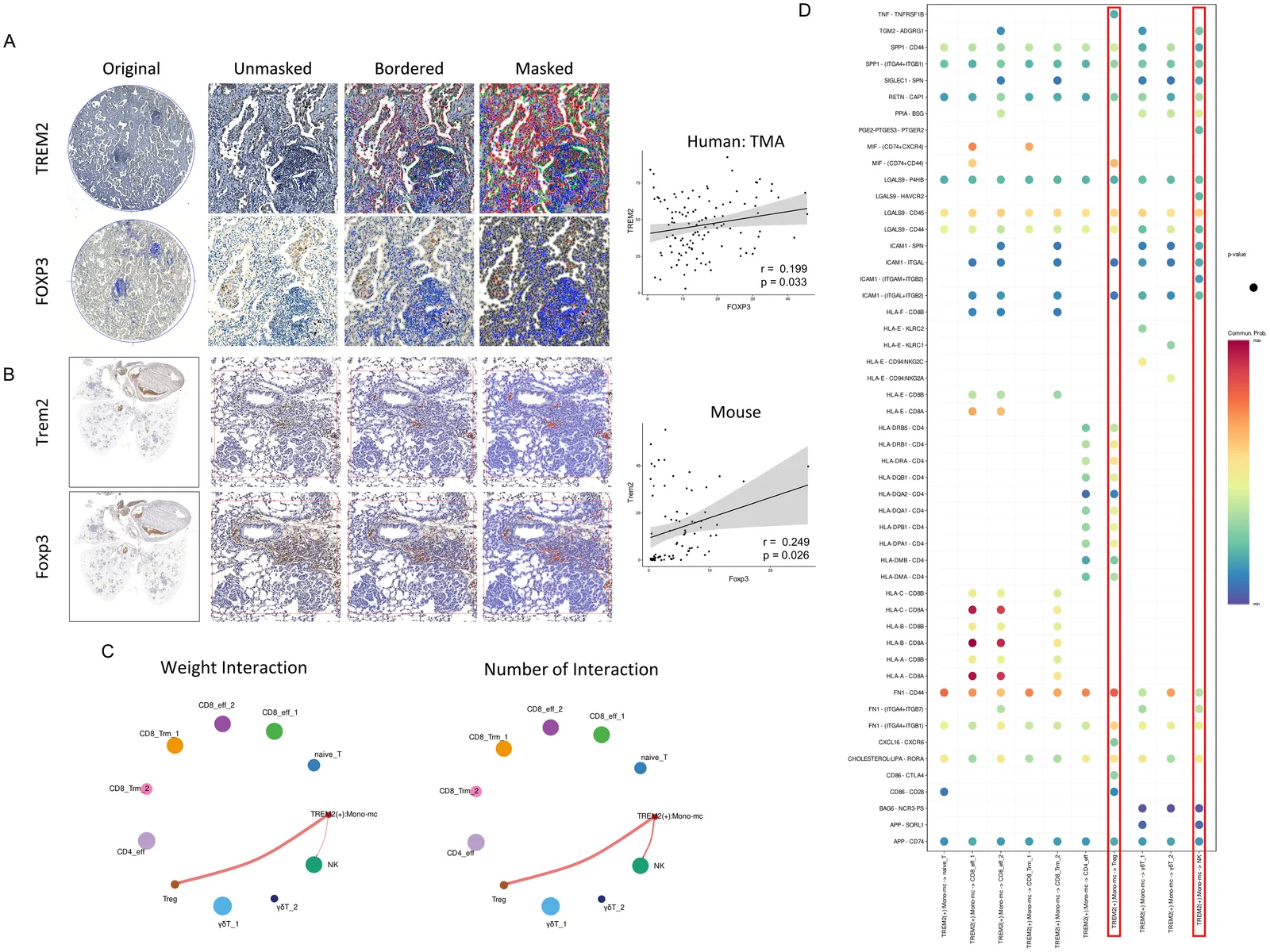
Quantitative association between TREM2^+^ TAMs and FOXP3^+^ cells. **(A)** Representative images and analysis workflow of immunohistochemistry (IHC) for TREM2 and FOXP3 in a tissue microarray (TMA) of 116 human lung adenocarcinomas. **(B)** Representative images and analysis workflow of IHC for Trem2 and Foxp3 in lung tissues from the LSL-Kras G12D mouse lung cancer model treated with vehicle or cisplatin. **(C)** Correlation plots showing the relationship between the numbers of TREM2^+^ and FOXP3^+^ cells in the human lung adenocarcinoma TMA (left) and in the LSL-Kras G12D mouse lung cancer model (right). **(D)** Caterpillar plots showing the weight of interaction (left) and the number of interactions (right) between TREM2^+^ TAMs and T/NK subclusters as inferred by CellChat analysis. **(E)** Dot plot showing candidate interacting molecules between TREM2^+^ TAMs and T/NK subclusters predicted by CellChat.

**Table 2:** Demographic characteristics of LUAD patients used for tissue microarray.

Parameters		Value
Age (mean ± SD)		66.8 ± 9.27
Gender	Male	57
	Female	59
Smoking Hx	Current	14
	Ex-smoker	22
	Non-smoker	80
Stage	I	54
	II	36
	IIIA	10
	IIIB	4
	IV	3
	na	9
Predominant histologic type	acinar	77
	lepidic	7
	micropapillary	2
	papillary	18
	solid	12

**Figure 3:**
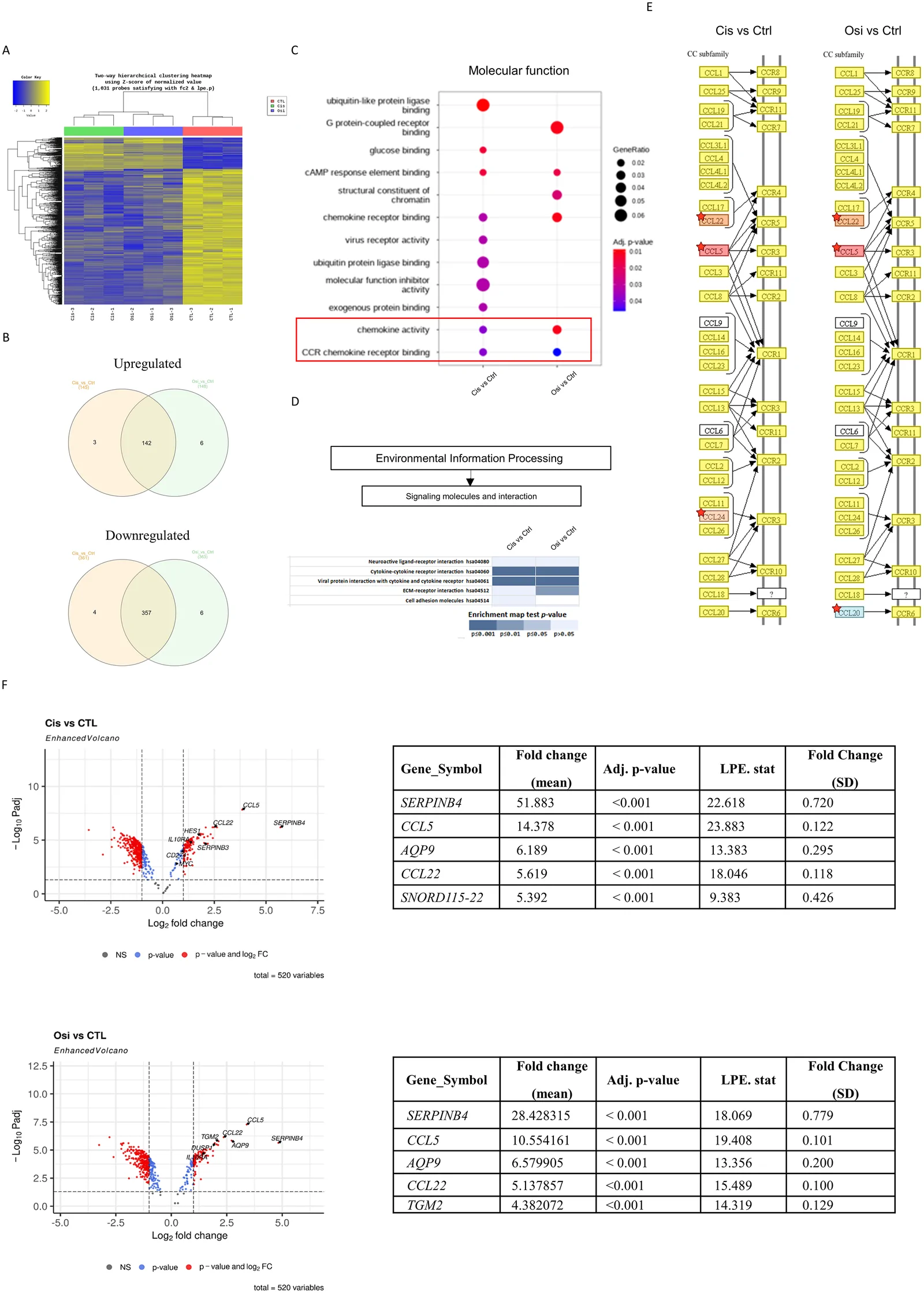
Microarray analysis of M0-polarized THP-1 cells treated with cisplatin or osimertinib. **(A)** Heatmap with hierarchical clustering of upregulated and downregulated genes obtained from microarray data of THP-1 cells differentiated to an M0 state by treatment with 150 nM PMA for 72 h and subsequently exposed to 500 nM cisplatin or 100 nM osimertinib for 16 h. **(B)** Venn diagram showing the overlap between the sets of upregulated and downregulated genes. **(C)** Dot plot of Gene Ontology molecular function terms identified by gene enrichment and functional annotation analysis using g:Profiler. **(D)** Enrichment map of KEGG pathways derived from the microarray data. **(E)** Schematic diagram depicting the predicted molecular interactions within the “cytokine–cytokine receptor interaction” pathway identified in **(D)**. **(F)** Volcano plots (left) showing upregulated genes in THP-1 cells treated with 500 nM cisplatin (top) or 100 nM osimertinib (bottom) for 16 h, and corresponding tables (right) listing the top five upregulated genes for each treatment condition.

### 3.3 TREM2^+^ TAMs influence the T-cell subset distribution

To explore the candidate mediators of this association, we interrogated the same scRNA-seq datasets using CellChat. The net interaction analysis indicated significant interactions specifically between TREM2^+^ TAMs and the Treg and NK subsets (**Figure 2D**; **Supplementary Figure 4**), and we used this result to nominate this interaction for further study (**Figure 2E**). Although the CCL5-CCR5 and CCL22-CCR4 pairs are present in the CellChat database (v2.2.0.9001), they were not returned as significant in these treatment-naïve data, in which the TREM2^+^ cluster expresses these ligands at low levels, consistent with a treatment-induced rather than constitutive axis. We therefore identified the candidate ligands downstream from the treatment-induced chemokine screen (below), and confirmed that the cognate receptors are available on Tregs. *CCR4* was largely restricted to CD4^+^ T cells including Tregs, whereas *CCR5* was broadly expressed across T-cell subsets including Tregs (**Supplementary Figure 5**). Of the ligands, *CCL22* showed higher expression in the myeloid compartment, whereas *CCL5* was expressed more broadly, including in cytotoxic T/NK cells, consistent with CCL22-CCR4 forming a directional myeloid→Treg signal and with CCL5-CCR5 being a multifunctional axis. To functionally validate chemotherapy-induced mediators, we performed gene expression microarray analysis on M0-polarized THP-1 Mφ treated with cisplatin or osimertinib. Both agents induced overlapping DEG sets (**Figures 3A, B****;** **Supplementary Tables 4**, **5**), with enrichment for leukocyte chemotaxis and CCR-related pathways (**Figures 3C, D**). Notably, *CCL5* and *CCL22* emerged among the top upregulated chemokines in both contexts (**Figures 3E, F**).

### 3.4 Expression of *CCL5* and *CCL22* is *TREM2*-dependent

We next asked whether chemotherapy-induced *CCL5* and *CCL22* upregulation depends on *TREM2*. To address this, we used two independent approaches: (i) genetic knockdown of *TREM2* and (ii) pharmacologic inhibition using the TREM2-inhibitory peptide IA9. In each approach, we quantified *CCL5/CCL22* expression. First, THP-1 cells bearing sh*TREM2* or a control shRNA were generated and polarized into M0/M1/M2 phenotypes, or exposed to cisplatin or osimertinib, followed by immunoblotting (**Figures 4A, B**) (20). In sh*TREM2* cells, TREM2 expression was not induced upon M2 polarization, and drug treatment of M0 cells did not increase TREM2. Among these shRNA-modified cells, treatment with cisplatin or osimertinib significantly upregulated *CCL5* and *CCL22* in the control cells, whereas this drug-induced increase was abrogated in *TREM2-*silenced cells (**Figure 4C**; **Supplementary Tables 6**, **7**; **Supplementary Figure 6A**). We analyzed the effects of IA9–a synthetic 9-mer peptide that disrupts the interaction between TREM2 and the adaptor DAP12 (TYROBP)–in M0-polarized THP-1 cells, followed by quantitative RT-qPCR to assess transcript levels (**Figure 4D**, **Supplementary Tables 8**, **9**; **Supplementary Figure 6B**). Consistent with the shRNA-mediated knockdown results, IA9 pretreatment suppressed the cisplatin- and osimertinib-induced upregulation of *CCL5* and *CCL22*.

**Figure 4:**
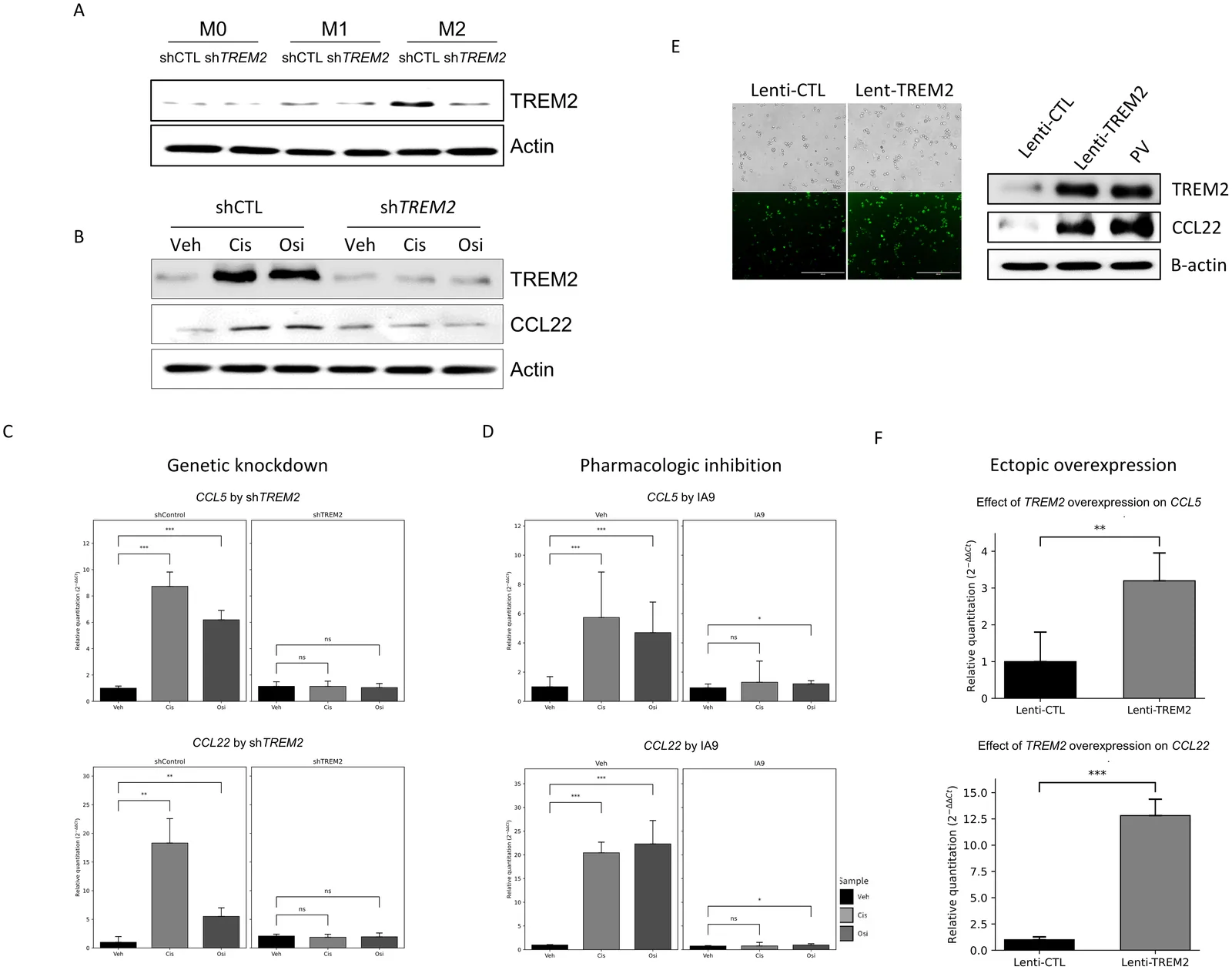
Effects of TREM2 inhibition (shRNA, IA9) and overexpression on *CCL22* and *CCL5* expression. **(A)** THP-1 cells transduced with a human *TREM2*–targeting shRNA (sh*TREM2*) or control shRNA (shCTL) were polarized to M0/M1/M2 and analyzed. Representative immunoblots for TREM2 and β-actin. **(B)** shCTL and sh*TREM*2 M0 cells were treated with cisplatin (500 nM) or osimertinib (100 nM) for 16 h, followed by immunoblotting (TREM2, CCL22, β-actin; representative). **(C)** RT-qPCR under the same conditions as in **(B)** for *CCL5* (top) and *CCL22* (bottom). Data are expressed as 2*^-ΔΔCt^*, normalized to *GAPDH*, and plotted relative to vehicle (veh). Within each cell line, Veh *vs*. Cis and Veh *vs.* Osi were compared using two-sided Welch’s t test. **(D)** M0 THP-1 cells were pretreated with TREM2 inhibitor, IA9 (1 µM for 1 h), and then exposed to cisplatin (500 nM) or osimertinib (100 nM) for 16 h. RT-qPCR quantification of *CCL5* and *CCL22* (normalization and statistics as in **(C)**). **(E)** THP-1 cells were transduced with control or *TREM2*-expressing lentivirus and examined by fluorescence microscopy (upper; scale bar, 200 µm) and immunoblotting (lower). **(F)** Baseline RT-qPCR in control *vs.* TREM2-overexpressing cells for *CCL5* (top) and *CCL22* (bottom). Analyses used two-sided Welch’s t test. Bar graphs show mean ± s.e.m. with biological replicates n = 3. Significance: *p < 0.05, **p < 0.01, ***p < 0.001. veh, vehicle; cis, cisplatin; osi, osimertinib; IA9, TREM2-inhibitory peptide; shCTL, control shRNA; sh*TREM2*, *TREM2* shRNA.

Finally, to evaluate whether TREM2 expression alone is sufficient for CCL5 and CCL22 induction, we analyzed THP-1 cells stably overexpressing *TREM2* via lentiviral transduction. Notably, *TREM2* overexpression alone robustly increased the mRNA levels of both *CCL5* and *CCL22* in the absence of chemotherapy (**Figures 4E, F**). *CCL22* upregulation was confirmed by immunoblotting, whereas *CCL5* was not reliably detected in our whole-cell lysate immunoblot conditions, likely because of its low molecular weight and secreted nature. In this saturated context, additional treatment with cisplatin or osimertinib did not further enhance chemokine mRNA expression beyond the already elevated baseline, suggesting that maximal induction had already been achieved by *TREM2* overexpression alone (**Supplementary Figure 7**; **Supplementary Tables 10**, **11**). Collectively, these data demonstrate that *TREM2* is sufficient to drive *CCL5*/*CCL22* upregulation to levels comparable with those induced by cisplatin or osimertinib, which is in line with our loss-of-function data showing that *TREM2* is required for drug-induced chemokine induction in TREM2^+^ TAMs.

### 3.5 Mechanism of *CCL5* and *CCL22* upregulation

Having established the *TREM2*-dependence of chemotherapy-induced chemokine induction, we next mapped the downstream pathways governing *CCL5* (and, in parallel, *CCL22*) expression. M0-polarized THP-1 macrophages were pretreated for 1 h with BAY 61-3606 (SYK), dasatinib (Src), AZD6244 (ERK), LY294002 (PI3K/AKT axis), ruxolitinib (JAK), STX-0119 (STAT3), or TPCA1 (NF-κB), followed by exposure to cisplatin or osimertinib.

In THP-1 cells (**Figure 5A**; **Supplementary Tables 12–15****),** cisplatin- and osimertinib-induced *CCL5* upregulation was suppressed by the inhibition of Src, NF-κB, and STAT3; however, SYK inhibition paradoxically increased *CCL5* in this context. For *CCL22* in THP-1 cells, cisplatin/osimertinib-driven induction was consistently attenuated by inhibition of SYK, Src, PI3K/AKT, STAT3, and NF-κB, whereas ERK or JAK inhibition showed no significant impact on *CCL22* levels.

**Figure 5:**
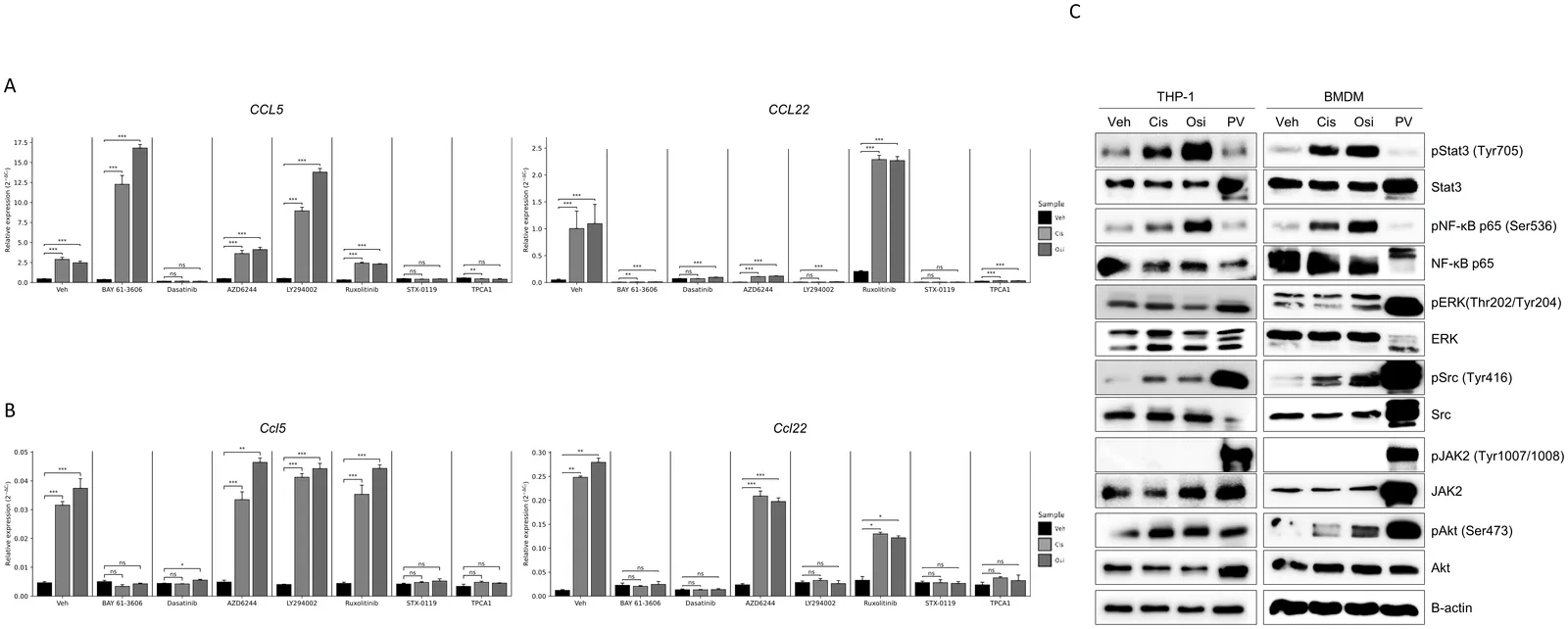
Signaling pathways regulating *CCL5* and *CCL22* expression. **(A)** RT-qPCR analysis of *CCL5* (left) and *CCL22* (right) mRNA expression in M0-polarized THP-1 cells pretreated for 1 h with (1) the SYK inhibitor BAY 61-3606 (100 nM), (2) the Src inhibitor dasatinib (30 nM), (3) the MEK inhibitor AZD6244 (500 nM), (4) the PI3K/Akt inhibitor LY294002 (10 μM), (5) the JAK inhibitor ruxolitinib (100 nM), (6) the STAT3 inhibitor STX-0119 (10 μM), or (7) the NF-κB inhibitor TPCA1 (2.5 μM), followed by stimulation with 500 nM cisplatin or 100 nM osimertinib for 16 h. Differences between vehicle and cisplatin or osimertinib within each pretreatment condition were evaluated using Welch’s t test. **(B)** RT-qPCR analysis of *Ccl5* (left) and *Ccl22* (right) mRNA expression in M0-polarized bone marrow–derived macrophages (BMDMs) treated with the same panel of inhibitors and cisplatin or osimertinib as in **(A)**. Differences between vehicle and cisplatin or osimertinib were evaluated using Welch’s t test. **(C)** Immunoblot analysis of M0-polarized THP-1 cells (left) and BMDMs (right) treated with 500 nM cisplatin, 100 nM osimertinib, or 2 nM PV (positive control) for 30 min. In **(A, B)** bar graphs show mean ± s.e.m. with biological replicates n = 3. Significance: *p < 0.05, **p < 0.01, ***p < 0.001. Abbreviations: Veh, vehicle; Cis, cisplatin; Osi, osimertinib; BMDM, bone marrow–derived macrophage; PV, pervanadate.

To extend these findings to primary murine macrophages, BMDMs derived from C57BL/6 mice were analyzed using the same inhibitor panel and chemotherapy exposure scheme. In BMDM M0 cells (**Figure 5B**; **Supplementary Tables 16–19****),** cisplatin- and osimertinib-induced *Ccl5* upregulation was suppressed by Src, NF-κB, and STAT3 blockade; notably, in contrast to THP-1 cells, SYK inhibition suppressed *Ccl5* in BMDMs, indicating cell type-dependent responses. For *Ccl22*, cisplatin/osimertinib-driven upregulation was consistently attenuated by the inhibition of SYK, Src, PI3K/AKT, STAT3, and NF-κB, whereas ERK or JAK inhibition did not reduce *Ccl22*.

Immunoblotting confirmed that chemotherapy activated the phosphorylation of STAT3, NF-κB, Src, and AKT, whereas JAK2 and ERK phosphorylation was not detectable under these conditions (**Figure 5C****).** Together, these data indicate that although *CCL5/Ccl5* and *CCL22/Ccl22* induction is dependent on *TREM2*, downstream signaling diverges: *CCL5/Ccl5* is independent of PI3K/AKT and relies on a TREM2–Src–NF-κB–STAT3 axis, with SYK-dependence varying by the cell type. Conversely, *CCL22/Ccl22* engages DAP12–SYK, PI3K/AKT, Src, NF-κB, and STAT3 signaling. This pathway selectivity suggests that *TREM2*-mediated immune modulation may be chemokine-specific, enabling targeted interference with CCR4/CCR5-directed recruitment programs in the TME.

### 3.6 *Trem2* inhibition attenuates cisplatin-driven Foxp3^+^ enrichment

To further evaluate how chemotherapy-induced *Trem2* signaling shapes the tumor microenvironment, we established a syngeneic orthotopic lung cancer model by injecting LLC cells into C57BL/6 mice. In a pilot experiment (four mice per group), mice were pretreated with IA9, a human TREM2–derived inhibitory peptide designed to disrupt TREM2–DAP12 interaction, or with vehicle and subsequently administered cisplatin. Lungs were harvested and subjected to Foxp3 immunohistochemistry (**Figure 6A**, quantified in **Figure 6B**; **Supplementary Table 20**), together with dual immunofluorescence for Foxp3 and CD3 confirming that the Foxp3^+^ cells were a T-cell subset (**Figure 6C**). Compared with vehicle-treated tumors, cisplatin-treated tumors showed increased infiltration of Foxp3^+^ cells, most prominently in the peri-necrotic and peri-apoptotic regions, and this cisplatin-associated enrichment was significantly attenuated by IA9 pretreatment (Cis vs. Cis+IA9, P < 0.001), restoring Foxp3^+^ infiltration to a level statistically indistinguishable from vehicle (Cis+IA9 *vs*. Veh, P = 0.982) (**Figure 6B****;** **Supplementary Table 20**). We next assessed drug-associated changes in Trem2^+^ cell infiltration and Ccl22/Ccl5 signals by immunofluorescence; in cisplatin-treated tumors, increased Trem2^+^ cells and enhanced Ccl22/Ccl5 signals co-localized predominantly around areas of chemotherapy-induced cell death, indicating that these chemokine signals are associated with Trem2^+^ cells (**Figure 6D**).

**Figure 6:**
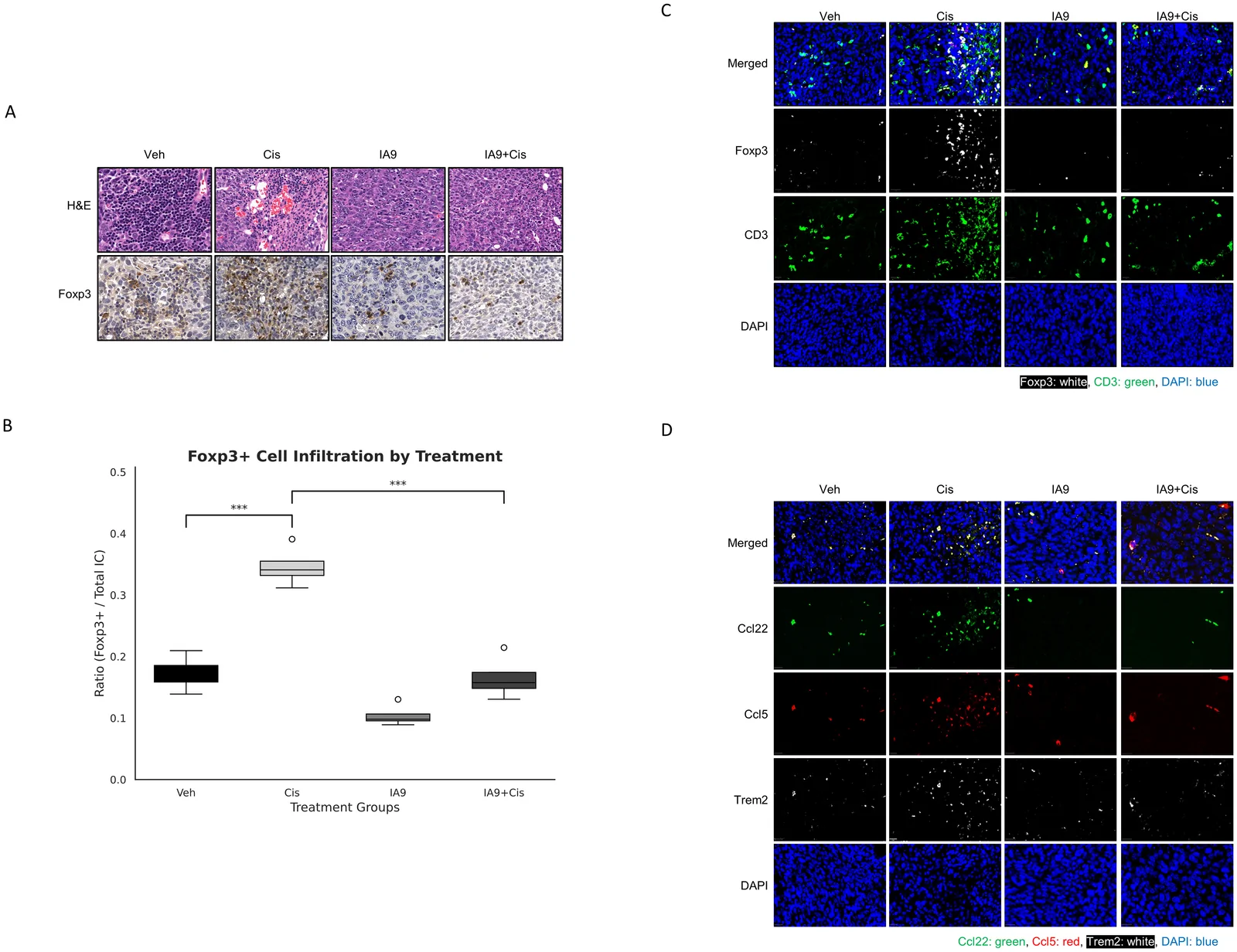
Trem2 inhibition attenuates cisplatin-driven Foxp3^+^ enrichment in an orthotopic lung cancer model. **(A)** Representative hematoxylin and eosin (H&E) staining and Foxp3 immunohistochemistry (IHC) of orthotopic Lewis lung carcinoma (LLC) tumors from C57BL/6 mice treated with vehicle (Veh), cisplatin (Cis), IA9, or Cis + IA9. Representative images are shown; n = 4 mice per group. Scale bar, 20 µm. **(B)** Box-and-whisker plot denoting the fraction of Foxp3^+^ cells among total mononuclear cells, quantified using QuPath. Statistical significance was determined by one-way ANOVA followed by Tukey’s HSD *post-hoc* test; full pairwise comparisons are provided in **Supplementary Table 20**. **(C)** Representative immunofluorescence (IF) staining for Foxp3 (white), CD3 (green), and DAPI (blue) in orthotopic LLC tumors from the indicated treatment groups (Veh, Cis, IA9, Cis + IA9), confirming that Foxp3^+^ cells represent a CD3^+^ T-cell subset. Images are representative of n = 4 mice per group. Scale bar, 20 µm. **(D)** Representative IF staining for Ccl22 (green), Ccl5 (red), Trem2 (white), and DAPI (blue) in orthotopic LLC tumors from the indicated treatment groups, showing co-localization of Ccl22/Ccl5 signals with Trem2^+^ cells. Images are representative of n = 4 mice per group. Scale bar, 20 µm.

Collectively, these pilot *in vivo* findings suggest that chemotherapy can promote a *Trem2*-associated, Foxp3^+^ T-cell–enriched microenvironment, and IA9 treatment may blunt this response. In this orthotopic LLC model, IA9 pretreatment reduced Foxp3^+^ cells within the CD3^+^ T-cell compartment (**Figures 6A–C**) and attenuated cisplatin-associated increases in Trem2^+^ cells and *Ccl22/Ccl5* signals in the TME (**Figure 6D**), suggesting a reversal of the chemotherapy-induced immunosuppressive niche.

## 4 Discussion

Cytotoxic chemotherapy and targeted therapies are known to remodel the TME; however, the myeloid cell-centered program linking drug exposure and secondary immunosuppression remains elusive. In this study, we identified TREM2/Trem2^+^ TAMs as a mechanistic bridge between treatment and Treg accumulation in NSCLC. By integrating findings from human NSCLC, murine models, and functional assays, we showed that therapy engages baseline *TREM2/Trem2* signaling in TAMs to drive chemokine output, *CCL22/Ccl22* and *CCL5/Ccl5*. This process facilitates the CCR4/CCR5-directed influx of Tregs, thereby establishing an immune-evasive niche.

Several convergent lines of evidence underpin this interpretation. First, in human NSCLC and a *Kras*-driven lung cancer model, tumors were concurrently enriched for TREM2^+^/Trem2^+^ and FOXP3^+^/Foxp3^+^ cells relative to those in matched normal lungs, and their densities were positively correlated. Although the tissue-level correlation coefficients were modest (r ≈ 0.20–0.25), as expected given the compositional heterogeneity of the TME, the direction was reproduced by three independent approaches — human tissue, a Kras-driven model, and aggregated single-cell data — and was further corroborated by categorical between-group comparisons in an independent cohort, in which the TREM2^+^ fraction was higher in tumor than normal lung and was increased by cisplatin (20). A public LUAD cohort likewise showed a positive TREM2–FOXP3 mRNA association. We therefore interpret this relationship as modest but consistent, and note that our conclusions rest primarily on the functional perturbation data rather than on correlation magnitude. Second, in M0-polarized macrophage systems, both cisplatin and osimertinib induced robust increases in *CCL22*/*CCL5* (THP-1) and *Ccl22*/*Ccl5* (murine BMDMs) transcripts. Third, genetic perturbation of *TREM2* in THP-1 cells abrogated drug-induced chemokine induction, and IA9 treatment similarly suppressed this response. Conversely, *TREM2* overexpression alone was sufficient to elevate the chemokine transcripts and establish a high baseline that was not further amplified by drug exposure. Collectively, these findings suggest that chemotherapy and targeted therapy predominantly leverage the pre-existing *TREM2/Trem2* signaling capacity, rather than requiring overt drug-driven upregulation of TREM2/Trem2 protein, to enable chemokine induction in TREM2^+^ TAMs (16, 23).

Pathway dissection suggests that the downstream wiring for chemokine induction diverges. In both THP-1 M0 cells and murine BMDMs, inhibitor studies and phospho-immunoblotting converged on the SYK–PI3K/AKT–Src–NF-κB–STAT3 axis as the principal route to *CCL22*/*Ccl22* transcript induction following chemotherapy, whereas JAK and ERK blockade did not diminish *CCL22*/*Ccl22*. In contrast, *CCL5*/*Ccl5* transcript elevation was PI3K/AKT independent, relied on Src–NF-κB–STAT3, and showed cell-type–specific dependence on SYK, with SYK inhibition suppressing *Ccl5* in BMDMs but producing a paradoxical increase in *CCL5* in THP-1 cells. These data fit a TREM2–DAP12–ITAM framework in which proximal SYK activation fans out into multiple, context-sensitive nodes; however, the dominance of specific branches (e.g., PI3K/AKT versus Src) appears to be chemokine- and cell-of-origin–specific. Mechanistically, this divergence could reflect promoter architecture and transcription-factor dependencies: *CCL22* has PU.1- and NF-κB-rich promoter elements and resides within a chemokine cluster on 16q13, whereas *CCL5* on 17q11.2–q12 (24, 25) exhibits promoter polymorphisms that modulate NF-κB binding, together with differences in chromatin accessibility between primary macrophages and myelomonocytic cell lines.

Placing our findings in context, TREM2^+^ TAMs have been broadly linked to immune suppression and adverse outcomes across multiple tumor types (16, 19, 26). The present study extends this framework by reconstructing an actionable, treatment-evoked axis: chemotherapy/EGFR-TKI → *TREM2/Trem2*-dependent signaling in TREM2^+^ TAMs → induction of *CCL22/Ccl22* and *CCL5/Ccl5* transcripts → CCR4/CCR5-mediated recruitment of FOXP3^+^/Foxp3^+^ cells (16, 23). To our knowledge, this combination of single-cell mapping (21, 22), quantitative tissue correlations, loss- and gain-of-function perturbations of *TREM2/Trem2*, and pathway-level interference within the same study provides an integrated, causal chain from drug exposure to Treg-enabling chemokine programs. The observed pathway bifurcation also helps reconcile heterogeneous reports on *CCL5* in cancer, indicating that in a *TREM2/Trem2*-high, NF-κB/STAT3-dominant context, *CCL5/Ccl5* may preferentially support myeloid–Treg crosstalk and immune suppression (27–30). The cell-type–specific behavior of SYK is noteworthy. BMDMs retaining native ITAM-coupled receptor networks showed SYK-dependence for both *Ccl22* and *Ccl5*, aligning with canonical DAP12–SYK signaling downstream of *Trem2* (16, 31–33). In contrast, THP-1 cells showed a paradoxical increase in *CCL5* upon SYK inhibition, suggesting compensatory routing through the Src or PKC–MAPK modules, or alternative ITAM-independent inputs (33, 34). Although our results intentionally limit interpretation to the observed effects, these differences emphasize that myeloid ontogeny and differentiation state shape the topology of *TREM2/Trem2*-connected signaling and by extension, the efficacy of pathway-specific interventions.

Our study has limitations that inform the next steps. First, the ligands that engage *TREM2* under chemotherapy or EGFR-TKI exposure remain undefined; multiple endogenous or stress-associated ligands have been proposed, and *TREM2* cross-linking can activate downstream signaling, but whether cisplatin or osimertinib directly alters ligand availability or membrane microdomains remains unresolved. Second, although we used an orthotopic, immunocompetent model to prioritize intact host immunity and avoid on-target cytotoxicity confounders in *EGFR*-mutant tumor cells, this choice limits direct extrapolation to specific *EGFR* contexts. Moreover, the LLC line is a poorly differentiated lung carcinoma rather than an adenocarcinoma, so the *in vivo* findings should be extrapolated to LUAD with this histological difference in mind. Future work across engineered *EGFR*-mutant models will be important. Third, IA9 acts through a ligand-independent SCHOOL mechanism that disrupts TREM2–DAP12 interactions; despite supportive *in vivo* activity in prior reports, species-specific sequence differences warrant careful pharmacological and dose–response evaluation in lung cancer models. Fourth, the SYK-dependence discrepancy between THP-1 and BMDM for *CCL5* underscores the need for orthogonal validation in primary human macrophages and *in situ* systems, ideally with chromatin and transcription-factor occupancy analyses linking the signaling nodes to promoter-level control.

Notwithstanding these caveats, the data yield several translational implications. First, *TREM2* emerges as a tractable therapeutic node as genetic knockdown of *TREM2* and its peptide inhibition curtailed chemotherapy-induced chemokine transcripts (23). Second, the *CCR4/CCR5* axes downstream of *CCL22/CCL5* provide additional therapeutic targets to block the recruitment of FOXP3^+^ cells. The receptor-level data further refine this axis. In the scRNA-seq dataset, *CCR4* was largely confined to CD4^+^ T cells, including tumor-infiltrating Tregs, whereas the ligand *CCL22* was predominantly expressed in the myeloid compartment. This directional, compartmentalized configuration closely mirrors the CCL22–CCR4 axis, established as a primary driver of Treg trafficking in LUAD (35–37). The CCL5–CCR5 axis is less Treg-restricted: *CCL5* is constitutively expressed by cytotoxic CD8/γδT/NK cells and *CCR5* is broadly distributed across T-cell subsets, so this axis is multifunctional rather than Treg-specific. We therefore interpret *CCL5* as an additional, treatment-induced contributor to CCR5^+^ Treg recruitment operating alongside the more Treg-selective CCL22–CCR4 axis, consistent with the report on *CCR5*-dependent Treg homing to tumors (28). Because these chemokines are induced by anticancer treatment rather than constitutively expressed, this circuit represents a therapy-evoked, receptor-defined route for Treg enrichment that may be targetable downstream of TREM2. Third, our pathway map suggests rational combinations, indicating that inhibitors of Src, NF-κB, or STAT3 may suppress both chemokines, whereas PI3K/AKT blockade may reduce *CCL22* but spare *CCL5*, enabling chemokine-selective tuning. Fourth, the described myeloid–Treg chemokine program could inform biomarker strategies; for instance, baseline TREM2 in myeloid cells and on-treatment *CCL22*/*CCL5* transcripts could help stratify patients at risk for post-chemotherapy immune evasion and guide the timing of TREM2- or CCR4/CCR5-targeted therapies (16, 23).

In summary, our work delineates a treatment-evoked, *TREM2*-dependent chemokine circuit that links macrophage-lineage signaling to FOXP3^+^ cell recruitment in NSCLC. Chemotherapy and an EGFR-TKI induce *CCL22* and *CCL5* transcripts in TREM2^+^ TAMs in a TREM2-dependent manner, with *CCL22* relying on SYK–PI3K/AKT–Src–NF-κB–STAT3 and *CCL5* relying on Src–NF-κB–STAT3 with cell-type–specific SYK inputs. These findings provide a mechanistic basis for combination strategies that pair cytotoxic or targeted treatment with TREM2 blockade or CCR4/CCR5 axis inhibition, highlighting chemokine-specific circuitry as a lever to reshape the TME into an immune-supportive landscape.

## Data Availability

The microarray data generated in this study have been deposited in the Gene Expression Omnibus (GEO) under accession number GSE309879. The scRNA-seq datasets analyzed during the current study were obtained from previously published studies (21, 22). The complete analysis code for myeloid and T/NK subset identification, annotation, and downstream analyses is openly available at https://github.com/mbgld/pDC and https://github.com/mbgld/TREM_TNK. Additional data supporting the findings of this study are available from the corresponding author upon reasonable request.
