# Supplementary material for: TREM2+ macrophages mediate anticancer therapy-induced immune evasion in lung adenocarcinoma via the CCL5/CCL22-Treg axis
Source: Front Immunol. 2026 Oct 6;17:1914462. doi: 10.3389/fimmu.2026.1914462 (PMC13680435; doi:10.3389/fimmu.2026.1914462)
Supplement: Supplementary file 1 [file SupplementaryFile1.docx]

Supplementary Methods

# 1 Supplementary Data

## 1.1 Identification and Annotation of Myeloid Subsets

The single-cell RNA sequencing datasets (References 21, 22) were processed with the annotation pipeline previously established for this cohort (References 20, 22). All analysis code and parameters are openly available at <https://github.com/mbgld/pDC> and <https://github.com/mbgld/TREM_TNK>. Quality control and pre-processing. Doublets were removed with DoubletFinder (v2.0.3), and cells with a mitochondrial read fraction exceeding 10% or fewer than 200 detected genes were excluded (Seurat). Datasets were then integrated, and malignant epithelial cells were identified and removed using inferCNV (v1.12.0). Clustering and isolation of the myeloid compartment. All cells of the lung tumor microenvironment were clustered on a shared-nearest-neighbor graph (Seurat FindNeighbors, top 20 principal components) followed by the Louvain algorithm (FindClusters), and were assigned to major lineages. The myeloid compartment was isolated using canonical myeloid markers (*CD68, CSF1R, LYZ, AIF1*). Myeloid subclustering and annotation. The isolated myeloid cells were re-clustered (FindNeighbors, top 20 principal components; FindClusters, resolution 0.6), yielding a reproducible separation into 17 subclusters comprising four monocyte/differentiation states, nine macrophage states, and four dendritic-cell states. Subcluster markers were obtained with FindAllMarkers/FindMarkers (Wilcoxon rank-sum test, Bonferroni-adjusted P). The annotation followed the marker sets reported for this cohort (Reference 22: Results section 3.3 "Subclustering of MY cells"; Figure 2D; Supplementary Table 5). Representative markers were as follows:

- Monocyte/differentiation states — Mo-lineage; FCN1-mono (*FCN1*); Mono-Mc (*IL1B, S100A8, MRC1, C1QB*); CD163/LGMN (*CD163, LGMN*).
- Macrophage states — alveolar macrophages Alv-Mc1–8 (*MARCO, FABP4, PPARG*) and proliferating macrophages Prol-Mc (*MKI67, TOP2A*).
- Dendritic-cell states — cDC1 (*CLEC9A, CADM1*); cDC2 (*LAMP3, LY75*); monocyte-derived DC (mo-DC; *CD1C, FCER1A, CLEC10A*); plasmacytoid DC (pDC; *CLEC4C, LILRA4, TLR9*).

Definition of TREM2⁺ tumor-associated macrophages. After annotation, TREM2-high cells were extracted with TREM2.cell <- WhichCells(MY.sfj, expression = TREM2 > 2.5) and defined as one additional subcluster, following Reference 20. Their spatial mapping on the myeloid manifold, marker basis, and discriminating signature are described in the main text (Results 3.1) and Supplementary Table 2. Cross-cohort reproducibility of this state was supported by a conserved 40-gene tumor-enriched myeloid signature (including TREM2) shared across three independent datasets (Reference 20, Figure 1D).

Distribution and interaction analysis. Differences in subcluster distribution between normal-appearing and tumor tissue were assessed by dividing each subcluster's cell count by the per-case total and compared with the unpaired Wilcoxon rank-sum test. Intercellular interactions between TREM2⁺ TAMs and T/NK subclusters were estimated with CellChat(v2.2.0.9001).

## 1.2 RT-qPCR Data Processing

Quantitative PCR data were analyzed using the comparative *Ct* method. In multi-plate experiments (e.g., signaling inhibitor screens in THP-1 cells), ordinary least squares (OLS) regression models were used to account for technical variation*: ΔCt ~ Drug + C(Plate)*. To examine the suppression of drug-induced chemokine induction, we utilized a Difference-in-Differences (DiD) framework within the linear model: *ΔCt ~ Pretreatment X Drug + C(Plate)*. Heteroskedasticity-robust standard errors (HC3) were applied to ensure a valid inference. For single-plate experiments (e.g., BMDM assays), technical effect adjustments were omitted. Interaction coefficients were defined as [(*Drug - Veh)_Inhibitor_ - (Drug - Veh)_Vehicle_*]; a positive coefficient indicated significant suppression of drug-induced induction. Multiple testing across various inhibitors was controlled using the Benjamini–Hochberg false discovery rate (FDR), reported as q values.

## 1.3 Macrophage Differentiation and Polarization

### 1.3.1 THP-1–Derived Macrophages

THP-1 cells were cultured in RPMI-1640 supplemented with 10% FBS. To induce M0 macrophage differentiation, cells were treated with 150 nM phorbol 12-myristate 13-acetate (PMA; Sigma-Aldrich) for 24 h. M1 polarization was achieved by further culturing M0 cells in 10% RPMI medium containing 10 pg/mL LPS and 20 ng/mL IFN-γ. For M2 polarization, M0 cells were incubated with 20 ng/mL IL-4 and 20 ng/mL IL-13.

### 1.3.2 Mouse Bone Marrow-Derived Macrophages (BMDMs)

Primary BMDMs were isolated from 8- to 12-week-old C57BL/6 mice. Briefly, femurs and tibias were aseptically harvested, and the bone marrow was flushed with PBS using a 10-mL syringe. The cell suspension was passed through a 40 µm cell strainer and treated with RBC lysis buffer for 5 min. Viable cells were counted and seeded at a density of 1.5 × 10^6^ cells/mL in 6-well plates. Differentiation was induced by culturing cells in DMEM containing 10% FBS, 1% penicillin–streptomycin, and 20 ng/mL M-CSF for 6 days. The resulting mature BMDMs were used for subsequent experimental assays on day 6.

## 1.4 Genetic Manipulation of TREM2

### 1.4.1 shRNA-Mediated *TREM2* Knockdown

THP-1 cells were seeded in 6-well plates at a density of 3 × 10^5^ cells/well. Transfection was performed using the Origene TREM2 Human shRNA Plasmid Kit (Cat. No. TF300861) and Lipofectamine 3000. Briefly, 1 µg of shRNA plasmid DNA and P3000 reagent were diluted in Opti-MEM and mixed with Lipofectamine 3000 at a 1:1 ratio. After 15 min of incubation at room temperature, the complexes were added to cells maintained in Opti-MEM. After 48 h, the medium was replaced with RPMI-1640 containing 10% FBS and 0.5 µg/mL puromycin for stable cell selection.

### 1.4.2 Lentiviral Overexpression of *TREM2*

THP-1 cells were seeded in 96-well plates at 5,000 cells/well. On the following day, cells were transduced with TREM2 Human Tagged ORF Clone Lentiviral Particles (RC221132L4V; Origene) or control particles at an MOI of 60, supplemented with 8 µg/mL Polybrene. Following a 48 h incubation, cells were cultured in a growth medium containing puromycin to enrich for stably transduced cells.

## 1.5 IHC/ICC and Digital Image Analysis

### 1.5.1 Immunostaining Procedure

Formalin-fixed, paraffin-embedded (FFPE) tissue sections were deparaffinized in xylene and rehydrated. Antigen retrieval was performed in 0.1 M citrate buffer (pH 6.0) via boiling in a microwave. Endogenous peroxidase activity was quenched using 3% hydrogen peroxide. Slides were incubated with primary antibodies followed by HRP-conjugated or fluorophore-conjugated secondary antibodies. For IF, TrueVIEW Autofluorescence Quenching Kit (Vector Laboratories) was used, and slides were mounted with ProLong Gold Antifade Mountant with DAPI (Thermo Fisher Scientific). Images were captured as CZI files using an AxioScan 7 or LSM980 (Carl Zeiss).

### 1.5.2 QuPath-Based Quantification

Whole-slide images were analyzed using QuPath (v0.5.1). For automated cell detection, the nucleus detection function was utilized with a nucleus background radius set to 6.0 µm for human tissues and 5.0 µm for mouse tissues. Human tissue microarrays (TMAs) were analyzed by grouping cores by case, whereas mouse lung sections were assessed using 10 consistently labeled regions of interest (ROIs) per animal. To distinguish cell populations, an object classifier was trained using at least 100 manual annotations per cell class (e.g., TREM2+ for cytoplasmic/membranous staining and FOXP3+ for nuclear staining). The trained classifier was then applied to all sections, and the resulting counts and percentages were exported for statistical analysis.

## 1.6 Orthotopic Injection and Animal Monitoring

### 1.6.1 Tumor Cell Implantation

Eight-week-old C57BL/6 mice were maintained in a specific pathogen-free (SPF) facility under a 12-h light/dark cycle. LLC cells were prepared at a density of 5 × 10^6^ cells/mL in a 1:1 mixture of PBS and Matrigel. Under anesthesia (induction: 3–4%; maintenance: 1.5–2% isoflurane), a 31-gauge needle (BD 328822) was inserted 5 mm deep into the left lung through the 5^th^ intercostal space to deliver 20 µl of the cell suspension (~ 1 × 10^5^ cells/mouse).

### 1.6.2 Monitoring and Endpoints

Animals were allowed to recover on a warming pad and were monitored twice weekly for clinical signs, including body weight, respiratory patterns, and behavioral changes. Humane endpoints were predefined as weight loss ≥ 20% or overt signs of respiratory distress. Upon meeting these criteria, mice were euthanized, and lungs were harvested (Supplementary figure 1). Group allocation was randomized, and investigators were blinded to the treatment groups during outcome assessment. Exclusion criteria included peri-procedural mortality or technical failure (e.g., pneumothorax confirmed by immediate post-procedural observation).
