# Supplementary material for: TREM2+ macrophages mediate anticancer therapy-induced immune evasion in lung adenocarcinoma via the CCL5/CCL22-Treg axis
Source: Front Immunol. 2026 Oct 6;17:1914462. doi: 10.3389/fimmu.2026.1914462 (PMC13680435; doi:10.3389/fimmu.2026.1914462)
Supplement: Supplementary file 2 [file SupplementaryFile2.docx]

**Supplementary Figure Legends**

**Supplementary Figure 1. Experimental design of the orthotopic lung tumor mouse model.** Eight-week-old C57BL/6 mice were allowed to stabilize for one week prior to tumor implantation. 1 X 10^5^ Lewis lung carcinoma (LLC) cells, suspended in a 1:1 mixture of PBS and Matrigel, were orthotopically injected into the left 5^th^ intercostal space using a 31-gauge needle insulin syringe. One-week post-injection, mice were randomly assigned to four treatment groups. Eight days after starting the indicated drug treatment, the mice were euthanized and their lungs were harvested for subsequent molecular and histological analyses.

**Supplementary Figure 2. Box plots comparing the proportions of individual T/NK subclusters between lung cancer (Tu) and adjacent normal-like lung (NL), excluding the Treg subcluster shown in Figure 1D.** P-values were calculated using the Wilcoxon signed-rank test.

**Supplementary Figure 3. Whole-slide scan of immunohistochemistry (IHC) for TREM2 and FOXP3 in the tissue microarray (TMA) comprising 116 human lung adenocarcinomas, from which the representative high-magnification images shown in Figure 2A were obtained.**

**Supplementary Figure 4. Caterpillar plots showing the weight of interactions inferred by CellChat between each signal-sending T/NK subcluster or TREM2⁺ cells and the remaining T/NK subclusters.** The identity of the signal-sending cluster is indicated at the top of each panel.

**Supplementary Figure 5. Expression of *CCL22, CCR4, CCL5*, and *CCR5* across myeloid and T/NK subclusters in the treatment-naïve lung adenocarcinoma TME (scRNA-seq).**

Each panel shows a UMAP feature plot (top) and a violin plot (bottom) for (A) *CCL22*, (B) *CCR4*, (C) *CCL5*, and (D) *CCR5*. Values are log-normalized gene expression across individual cells; violin plots display the distribution per subcluster.

**Supplementary Figure 6. Suppression of drug-induced *CCL5* (top) and *CCL22* (bottom) expression by TREM2 inhibition.**

(A) THP-1 cells with *TREM2* knockdown (sh*TREM2*) or control shRNA (shControl) were treated with cisplatin (500 nM; left panels) or osimertinib (100 nM; right panels) for 16 h.

(B) M0-polarized THP-1 cells were pretreated with the TREM2 inhibitor IA9 (1 μM) or vehicle for 1 h, followed by exposure to cisplatin (500 nM; left panels) or osimertinib (100 nM; right panels) for 16 h.

mRNA levels of target genes were determined by qPCR and normalized to GAPDH. Data are expressed as log_2_ fold change relative to the vehicle control within each pretreatment group. Bars indicate the mean; error bars represent the 95% confidence interval (CI); dots denote individual wells (n=3). Statistical significance was determined by Welch’s t-test for between-group comparisons within each drug treatment. *p < 0.05, **p < 0.01, ***p < 0.001. Abbreviations: Veh, vehicle; Cis, cisplatin; Osi, osimertinib.

**Supplementary Figure 7. Effect of *TREM2* overexpression on drug-induced *CCL5* and *CCL22* expression.**

mRNA expression of *CCL5* (A) and *CCL22* (B) was analyzed in THP-1 cells overexpressing TREM2 (Lenti-TREM2) or control vector (Lenti-CTL) following treatment with cisplatin (500 nM) or osimertinib (100 nM) for 16 h. Target gene expression was determined by qPCR and normalized to GAPDH. Data are presented as log_2_ fold changes relative to the vehicle control. For each pretreatment group, log_2_ fold changes were calculated using WellRank-based pairing between drug-treated and vehicle-treated samples. Bars indicate the mean; error bars represent 95% confidence intervals (CIs); dots denote individual wells (n=3). Statistical significance between Lenti-CTL and Lenti-TREM2 groups for each drug was determined by Welch’s t-test. *p < 0.05, **p < 0.01, ***p < 0.001. Abbreviations: Veh, vehicle; Cis, cisplatin; Osi, osimertinib.
